# Calixarene-Based Supramolecular Sensor Array for Pesticide Discrimination

**DOI:** 10.3390/s24123743

**Published:** 2024-06-08

**Authors:** Yeye Chen, Jia-Hong Tian, Han-Wen Tian, Rong Ma, Ze-Han Wang, Yu-Chen Pan, Xin-Yue Hu, Dong-Sheng Guo

**Affiliations:** College of Chemistry, State Key Laboratory of Elemento-Organic Chemistry, Key Laboratory of Functional Polymer Materials (Ministry of Education), Frontiers Science Center for New Organic Matter, Collaborative Innovation Center of Chemical Science and Engineering, Nankai University, Tianjin 300071, China

**Keywords:** pesticide, sensor array, supramolecular chemistry, calixarene, discrimination

## Abstract

The identification and detection of pesticides is crucial to protecting both the environment and human health. However, it can be challenging to conveniently and rapidly differentiate between different types of pesticides. We developed a supramolecular fluorescent sensor array, in which calixarenes with broad-spectrum encapsulation capacity served as recognition receptors. The sensor array exhibits distinct fluorescence change patterns for seven tested pesticides, encompassing herbicides, insecticides, and fungicides. With a reaction time of just three minutes, the sensor array proves to be a rapid and efficient tool for the discrimination of pesticides. Furthermore, this supramolecular sensing approach can be easily extended to enable real-time and on-site visual detection of varying concentrations of imazalil using a smartphone with a color scanning application. This work not only provides a simple and effective method for pesticide identification and quantification, but also offers a versatile and advantageous platform for the recognition of other analytes in relevant fields.

## 1. Introduction

Pesticides are extensively used in agriculture, forestry, and livestock production to safeguard crops against pests, weeds, and plant diseases. However, improper pesticide usage can lead to severe environmental issues and pose risks to public health. One consequence is the contamination of the food chain through residual pesticides present in crops, fruits, water, and other products, causing harm to humans at the top of the food chain [1,2,3]. Pesticides have been shown to be involved in the pathogenesis of Parkinson’s, Hodgkin’s, and Alzheimer’s disease, as well as neoplasia, oxidative stress, and various respiratory and reproductive disorders [4,5,6]. Therefore, accurate pesticide identification and detection hold significant importance in the domains of agricultural production, food safety, environmental protection, and international trade. State-of-the-art techniques such as gas chromatography–mass spectrometry [7,8], liquid chromatography–mass spectrometry [9,10], surface-enhanced Raman spectroscopy [11], and enzyme-linked immunosorbent assay [12,13,14], have been commonly employed for pesticide detection. These techniques fulfill the requirements for detecting trace analytes, including high sensitivity, reproducibility, and accuracy. However, they have drawbacks such as being time-consuming, expensive, and reliant on complex instrumentation and trained personnel, limiting their applicability for on-site inspections. The development of electrochemical sensors [15], fluorescent sensors [16], colorimetric sensors [17,18], etc., represents a novel strategy for monitoring pesticide contamination [19]. Hence, there is still a critical need to continue developing rapid, cost-effective, and reliable methods that can be utilized at the point-of-use for the identification and detection of pesticides, safeguarding public health and ecological safety.

In order to differentiate closely related pesticide analytes, sensor arrays have gained popularity as a promising technology [20,21,22]. Each receptor in the array can interact with multiple analytes, but with varying binding affinity for each analyte [23,24]. The sensor array prioritizes pan-selectivity in its sensor units rather than highly specific selectivity, aiming to achieve improved cross-reactivity. Supramolecular macrocyclic hosts provide rich options for constructing sensor arrays, which are able to differentially encapsulate various pesticide molecules through non-covalent interactions [25]. To translate the molecular recognition process into a visual signal, indicator displacement analysis (IDA) has been widely employed in supramolecular sensing. The indicator guest is initially bound to the host, and the signal is modulated upon the competitive binding between the indicator and the analyte [23]. Among numerous signal transduction schemes, fluorescence methods are powerful sensing modalities due to their low cost, ease of use, and high sensitivity. Therefore, supramolecular fluorescent sensor arrays offer a promising solution to address rapid pesticide identification challenges.

**Figure 1:**
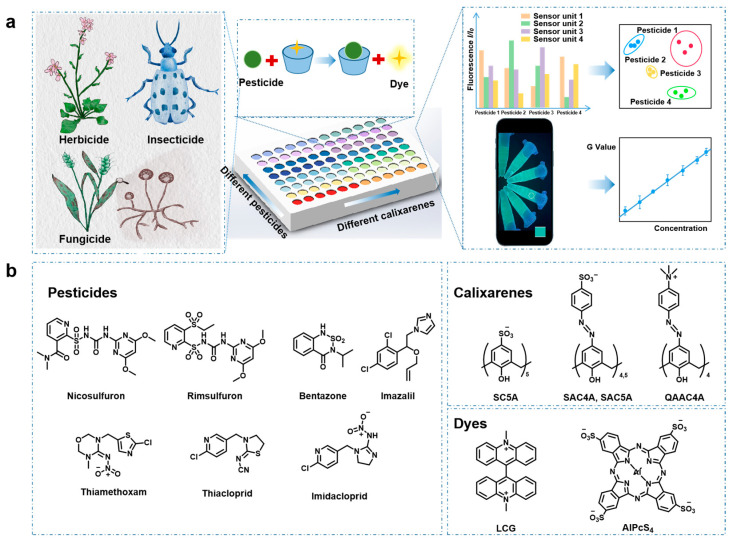
(**a**) Schematic representation of sensor array for pesticide discrimination. (**b**) Chemical structures of tested pesticides employed calixarenes (SC5A, SAC4A, SAC5A, and QAAC4A) and fluorescent dyes (LCG and AlPcS_4_).

In recent years, our research has focused on the molecular recognition and assembly properties of calixarenes and their application in disease diagnosis, drug delivery, and precise treatment. We have developed a toolbox of calixarene molecules [26,27]. Here, we selected four macrocyclic hosts from our calixarene library to provide the desired sensitive fluorescence detection of pesticides by IDA in aqueous media. These calixarenes possess different cavity sizes, depths, and upper-edge substituents, which are ideally complementary in their ability to recognize structurally similar pesticides. The fluorescence response of four calixarenes to each pesticide creates a unique fingerprint. Linear discriminant analysis (LDA) is a classical statistical approach for supervised dimensionality reduction, which is widely used in array-based sensing [28]. Through LDA, we successfully separated and distinguished seven representative pesticides, including insecticides, fungicides, and herbicides, on a plot even in the presence of soil extracts. The ability to differentiate among different concentrations of pesticides and binary mixtures of the sensor array has also been demonstrated. More interestingly, we showed the use of a smartphone color-scanning application for real-time monitoring of pesticide concentrations (Figure 1a).

## 2. Materials and Methods

### 2.1. Materials and Instruments

Nicosulfuron, rimsulfuron, imazalil, bentazone, thiamethoxam, thiacloprid, and imidacloprid were purchased from Shanghai Macklin Biochemical Co. Ltd., Shanghai, China. Soil extract was obtained from Beijing Solarbio Science&Technology Co. Ltd., Beijing, China. Lucigenin (LCG) was purchased from J&K Scientific, Beijing, China. Al (III) phthalocyanine chloride tetrasulfonic acid (AlPcS_4_) was obtained from Frontier Scientific, Inc., Philadelphia, PA, USA. The synthesis and characterization of sulfonatocalix[5]arene (SC5A), sulfonated azocalix[4]arene (SAC4A), sulfonated azocalix[5]arene (SAC5A), and quaternary-ammonium-modified azocalix[4]arene (QAAC4A) are provided in the Supplementary Information (Figures S1–S4). All aqueous solutions were prepared with ultrapure water from the Thermo Fisher Scientific (Waltham, MA, USA) purification system. Steady-state fluorescence spectra were recorded in a conventional quartz cell (light path 10 mm) on a Varian Cary Eclipse spectrometer (Agilent Technologies Inc., Santa Clara, CA, USA) equipped with a Varian Cary single-cell peltier accessory to control temperature.

### 2.2. Competitive Fluorescence Titration

The SC5A/LCG reporter pair solution was diluted into water (2.5 mL) in a quartz cuvette to give final concentrations at 1.0 μM/1.0 μM first. Then, the pesticide solution (800 μL at 0.1 mg mL^−1^) was added for competitive titrations. The spectra of fluorescence changes in report pairs caused by each pesticide were recorded.

### 2.3. Discrimination of Different Kinds of Pesticides

Two hundred microliters of reporter pair solution (2.0/2.0 μM) was added to a black 96-well plate, and fluorescence intensity (*I*_0_) was measured on a microplate reader. Then pesticide solution (30 μL at 0.1 mg mL^−1^) was introduced to the well, which was mixed and incubated at 25 °C for 3 min. The fluorescence intensity was measured and recorded as *I* at this time. Thus, the seven pesticides were tested against four reporter pairs 6 times to give a training matrix of 7 pesticides × 4 units × 6 replicates. Finally, the raw data matrix was handled using LDA in the Past 3 program.

### 2.4. Discrimination of Different Concentrations of Imazalil and Bentazone

Two hundred microliters of reporter pair solution (2.0/2.0 μM) was added to a black 96-well plate, and fluorescence intensity (*I*_0_) was measured on a microplate reader. Then imazalil solution was introduced to each well to give the final concentrations at 0, 0.1, 0.7, 1.0, 1.3, 2.6, 5.2, 7.8, 13.0, and 19.6 μg mL^−1^, respectively. The mixed solutions were incubated at 25 °C for 3 min, and then the fluorescence intensity (*I*) of each well was measured. Thus, different concentrations were tested against four reporter pairs 6 times to give a training matrix of 10 concentrations of imazalil (or 9 concentrations of bentazone at 0, 0.6, 2.6, 3.9, 7.8, 10.4, 26.1, 39.1, and 65.2 μg mL^−1^) × 4 units × 6 replicates.

### 2.5. Discrimination of Mixed Pesticides

Two hundred microliters of reporter pair solution (2.0/2.0 μM) was added to a black 96-well plate, and fluorescence intensity (*I*_0_) was measured on a microplate reader. Then the mixtures of nicosulfuron and rimsulfuron (30 μL, the total concentration was 1.0 mg mL^−1^ according to the proportion of 100%, 75%, 50%, 25%, and 0) or the mixtures of thiamethoxam and imidacloprid (30 μL, the total concentration was 0.5 mg mL^−1^ according to the proportion of 100%, 75%, 50%, and 0) were introduced to each well. The mixed solutions were incubated at 25 °C for 3 min, and then the fluorescence intensity (*I*) of each well was measured. Thus, each group of mixed pesticides was tested against four reporter pairs 6 times to give a training matrix of 4 mixtures × 4 units × 6 replicates.

### 2.6. Visual Determination of Imazalil

The SAC4A/LCG (2.0/2.0 µM) reporter pair was mixed up with various concentrations of imazalil for detection. The solution (1.2 mL) of each group was added into a polypropylene centrifuge tube, followed by excitation with a 254 nm handheld UV lamp. Then, color change could be taken by an iPhone 12, and the G-values of the red–green–blue (RGB) intensities in the images were extracted using WizEyes Tech G value was used to establish the calibration curve for the determination of imazalil. Error bars represent the mean ± s.d. (for *n* = 3 independent experiments).

## 3. Results and Discussion

### 3.1. Principle of Supramolecular Sensor Array

Seven representative pesticide analytes are shown in Figure 1b, of which nicosulfuron, rimsulfuron, and bentazone are herbicides, imazalil belongs to fungicides, while thiamethoxam, thiacloprid, and imidacloprid are insecticides. The above efficient pesticides are widely used in agricultural activities, but their intensive and incorrect application in crops results in contamination of the soil and product. For instance, neonicotinoids insecticides have a prolonged residual effect on soils [29]. Many in vitro and in vivo studies have shown their potential toxic effects on humans, including reproductive toxicity, neurotoxicity, hepatotoxicity/hepato carcinogenicity, immunotoxicity, and genetic toxicity. The three generations of neonicotinoids insecticides imidacloprid, thiamethoxam, and thiamethoxam are similar in chemical structure, but have different prices, activities, resistance levels, and persistence periods [30]. To distinguish these selected representative pesticides, our hosts of choice are sulfonic acid and quaternary ammonium-modified calixarenes, namely SC5A, SAC4A, SAC5A, and QAAC4A (Figure 1b). Differences in the upper edge modified group and cavity size of calixarenes provide variable binding affinity and differential signal output in array-based sensing, thereby endowing the sensor array with cross-reactivity. In addition, the introduction of the azo group expands the longitudinal cavity of the calixarene, which may result in higher binding affinity for the target analyte [31].

The IDA strategy was introduced for signal transduction. The principle of IDA sensing is that the fluorescent dye shows a large change in fluorescence, ideally a strong quenching or enhancement, upon binding to the host. The addition of analyte causes dye displacement, which results in regeneration of the intrinsic fluorescence of the dye [32]. IDA is compatible with differential sensing since arrays can be easily constructed from combinations of multiple receptors and multiple indicators [33]. Here, the negatively charged SC5A, SAC4A, and SAC5A (SCAs) and the positively charged QAAC4A as the macrocyclic hosts and the corresponding fluorescent dyes as the indicators (LCG for SCAs and AlPcS_4_ for QAAC4A) firstly form the complexes in the fluorescence-quenched state. As shown in Figure S5, the fluorescence of LCG and AlPcS_4_ was significantly quenched by the calixarene hosts to different extents. The complexation-induced quenching property of calixarenes was promising because the immediate displacement caused the “switch-on” sensing of analytes. The subsequent addition of various pesticide analytes to the fluorescence-off host-guest complexes resulted in varying degrees of recovery of the fluorescence signal as the pesticide bound to the receptor to release the indicator, which is ideal for differential sensing (Figure S6).

### 3.2. Discrimination of Pesticides by the Supramolecular Sensor Array

**Figure 2:**
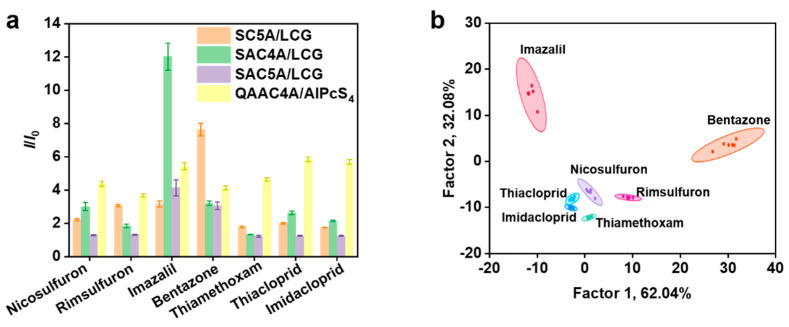
(**a**) Fluorescence response patterns of the sensor array ([calixarene] = 2.0 μM, [dye] = 2.0 μM) for different pesticides ([pesticide] = 13.0 μg mL^−1^). (**b**) Canonical score plot for the fluorescence response patterns determined by LDA with 95% confidence ellipses (*n* = 6).

On account of the structural similarity of some pesticides, one non-selective receptor may not show significant differences for a series of desired analytes, but an array of cross-reactive receptors is often found to display a unique fingerprint [34]. Hereon, the constructed sensor array consists of four fluorescent host-guest pairs for pesticide detection. For each sensor unit, the addition of different pesticides caused different changes in the fluorescence signal. For each pesticide, different fluorescence signal changes were induced by various supramolecular sensor units, which is a prerequisite for creating the unique pattern (Figure S6). To validate the feasibility of our proposed sensor array, we applied this four-element sensor array to seven pesticides in water. Corresponding to one pesticide and one reporter pair, we conducted six repeated experiments to generate a matrix of 7 pesticides × 4 units × 6 replicates (Table S1). As shown in Figure 2a, a fluorescence response pattern was constructed by collecting *I*/*I*_0_ values, where *I* and *I*_0_ denote the fluorescence intensity of the sensor unit in the presence and absence of pesticides, respectively. The four reporter pairs showed diverse fluorescence response signals against the seven pesticides, which should be attributed to the different interactions between calixarenes and analytes. This explicitly demonstrates the possibility of the constructed sensor array to identify pesticides.

LDA, a powerful statistical method extensively used in pattern recognition [28,35], was then used to transform the response patterns to the two-dimensional differential plot with 95% confidence ellipses (Figure 2b), which clearly shows that all of the seven pesticides are distinct from each other. The sensor array can discriminate among all the pesticides tested, and the jackknifed classification matrix with cross-validation revealed 100% accuracy, which proved the reliability of this chemical sensor array. Interestingly, the distribution of seven pesticides in the canonical score plot obviously associated with their categories, with neonicotinoids (thiamethoxam, thiacloprid, and imidacloprid) and sulfonylurea herbicides (nicosulfuron and rimsulfuron) having an approximate range, respectively, which is caused by their structural similarity. The performance of the fluorescent sensor array was further tested by distinguishing the blind samples. It was found that the identification accuracy of 20 blind samples at the level of 13.0 μg mL^−1^ was 95% (Table S2). The method proposed in this study is compared with other sensor arrays reported in the literature, as shown in Table S3. This supramolecular fluorescent sensor array demonstrates advantages in terms of the number of pesticide analytes and identification accuracy.

**Figure 3:**
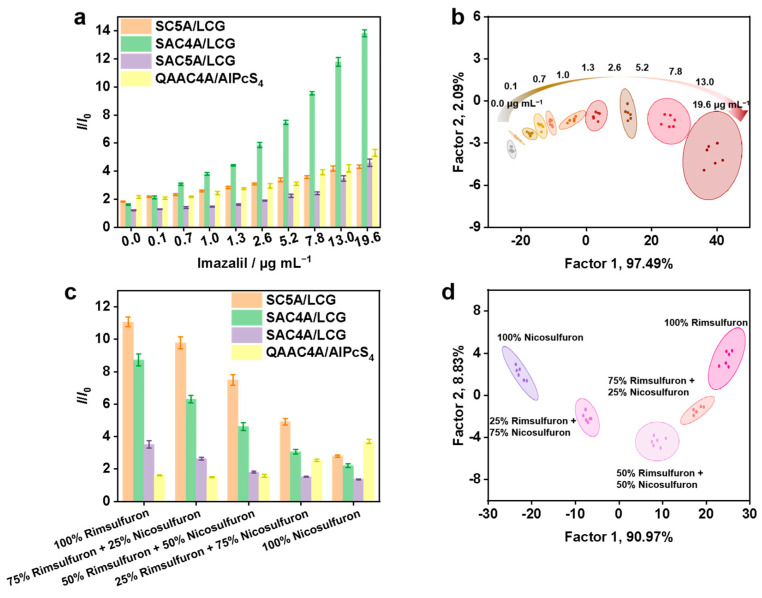
(**a**) Fluorescence response patterns and (**b**) canonical score plot for the detection of imazalil from 0–19.6 μg mL^−1^. Addition of solvent without imazalil resulted in an increase in optical path length, leading to a slight fluorescence response. (**c**) Fluorescence response patterns and (**d**) canonical score plot for mixtures (1.0 mg mL^−1^ for total concentration) of rimsulfuron and nicosulfuron.

After the successful qualitative identification of the pesticide, a semi-quantitative analysis was performed to further demonstrate the discriminatory ability of the sensor array. Long-lasting pesticides can persist in the soil, resulting in frequent incidents of crop damage, reduced yields, and even the extinction of certain crops. These issues have a significant impact on agricultural production, posing a serious threat to food security [36]. Imazalil was chosen as a representative pesticide because residues of such imidazole substances can be found in food commodities, implying a potential risk to human health due to their exposure toxicity [37]. As shown in Figure 3a,b, the sensor array can distinguish the concentration of 0–19.6 μg mL^−1^ of imazalil with good separation ability and perfect separation in a small concentration interval, which has great possibility for accurate quantitative detection of imazalil concentration. In addition, it showed a clear concentration dependence. As the concentration increases, the clusters are distributed from left to right along with factor 1, attributing to the recovery of fluorescence competition that is concentration dependent. Furthermore, the sensor array also showed good effects for discriminating bentazone with different concentrations (Figure S7), thereby demonstrating the universality of the sensor array. The success of semi-quantitative analysis makes it possible to realize monitoring of pesticide concentration.

Since usually mixtures of pesticides are used for crop protection, it is extremely worthwhile to be able to discriminate pesticide mixtures. Next, we verified the feasibility of distinguishing different ratios of the two mixed pesticides. Nicosulfuron and rimsulfuron were selected to test the sensitivity of the sensor array because both are sulfonylurea herbicides and have similar structures. In addition, nicosulfuron is the dominant species in the global sulfonylurea herbicide market, what is more, rimsulfuron is highly blendable, and DuPont has produced Ultim (nicosulfuron:rimsulfuron = 1:1) as a post-emergence blend, which is an important consideration in our choice of two pesticides. We employed the sensor array on binary mixtures at different ratios with a total concentration of 1.0 mg mL^−1^ (100% nicosulfuron, 75% nicosulfuron + 25% rimsulfuron, 50% nicosulfuron + 50% rimsulfuron, 25% nicosulfuron + 75% rimsulfuron, 100% rimsulfuron). To this aim, the response profiles of the array were recorded as shown in Figure 3c. The LDA score plot in Figure 3d for binary mixtures demonstrates that various mixtures are all well-clustered and separate enough from the groups of pure herbicides. The sensor array provided 100% cross-validation accuracy. In addition, we have made two mixtures of neonicotinoids, and the mixtures at certain ratios (100% thiamethoxam, 75% thiamethoxam + 25% imidacloprid, 50% thiamethoxam + 50% imidacloprid, and 100% imidacloprid) were prepared. It was found that different mixture ratios led to different fluorescence responses that were clearly separated from each other, as can be seen in the corresponding LDA score plot (Figure S8).

**Figure 4:**
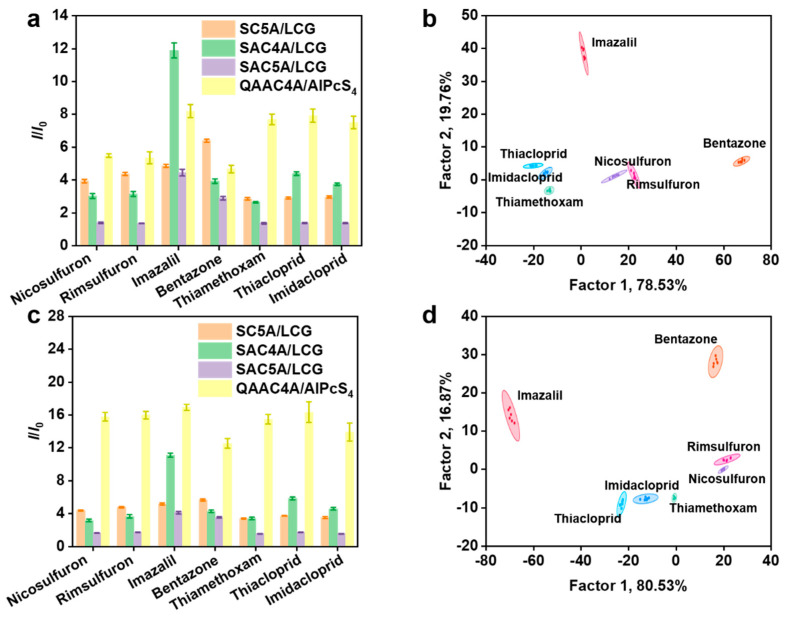
Fluorescence response patterns of the sensor array ([calixarene] = 2.0 μM, [dye] = 2.0 μM) for different pesticides in the presence of 10% (**a**), 20% soil extract (**c**). Canonical score plot for the fluorescence response patterns in the presence of 10% (**b**), 20% (**d**) soil extract determined by LDA with 95% confidence ellipses (*n* = 6).

Under practical circumstances, the presence of various inorganic and organic substances in the soil may potentially influence the identification of pesticide extracts. Therefore, to simulate the real situation as much as possible, we added soil extracts to the experimental setup, in which the induced interference may interact with the sensor units. Firstly, we introduced 10% (Figure 4a) and 20% (Figure 4c) of soil extracts. As expected, the sensor arrays reacted differently compared to the case without soil extract, and the fluorescence intensities of all the used reporter pairs increased. Even so, the LDA results showed that the seven tested pesticides were well clustered into seven groups with a classification accuracy of 100% (Figure 4b,d). Consequently, the results of the study indicate that the sensor array has resistance to external interference and has potential for practical applications.

### 3.3. Real-Time Scanometric Monitoring of Imazalil

**Figure 5:**
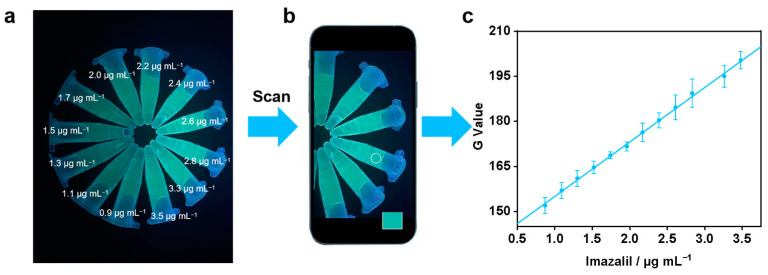
(**a**) The image of SAC4A/LCG (2.0/2.0 μM) with various concentrations (up to 3.5 μg mL^−1^) of imazalil taken by iPhone 12. (**b**) The images recorded by iPhone 12 with a color-scanning app. (**c**) Plots of G values against imazalil concentrations. G values are green color intensities directly scanned from WizEyes Tech.

To enable real-time/on-site pesticide monitoring in daily life, the fluorescence changes of the supramolecular sensing were converted into a visual detection mode by using a smartphone with an easy-to-access color scanning application (APP). In the semi-quantitative analysis for imazalil, the SAC4A/LCG sensor unit exhibited the most significant fluorescence change pattern. Therefore, we selected SAC4A/LCG as the fluorescent sensing reporter here. After contacting SAC4A/LCG with varying concentrations of carbendazim, LCG, which is competitively displaced due to the encapsulation of imazalil by SAC4A, emits fluorescence under a handheld UV lamp. The green color intensities (G values) of the fluorescent images could be read out easily from the APP (Figure 5a,b). G values in a certain range with the concentration of imazalil had a linear relationship (Figure 5c). The calibration curve was established, which can be used for quantitative analysis of imazalil. This demonstrates the potential of supramolecular sensing in practical applications. 

## 4. Conclusions

We have successfully achieved fluorescence discrimination of seven selected pesticides, even for those that are structurally similar or present in mixtures. This discrimination is based on the principle of IDA, where the competitive binding of pesticides triggers the fluorescence signal of the sensor unit. Furthermore, we have demonstrated the potential of supramolecular sensing for real-time and on-site detection by utilizing a color scanning program on a smartphone for visual and quantitative analysis of the pesticides. The present sensing approach exhibits the advantages of low cost, simple construction, and rapid response, which may pave the way towards portable sensor arrays for monitoring pesticides in complex matrixes to guarantee a friendly environment and food safety. Considering that there is room for improvement in sensitivity and accuracy, our future work aims to expand the library of calixarenes and integrate with the increasingly popular machine learning techniques to facilitate the precise discrimination of a wider range of pesticide types at lower concentrations.

## Data Availability

The datasets generated for this study are available on request to the corresponding author.

## Supplementary Materials

The supporting information can be downloaded at: https://www.mdpi.com/article/10.3390/s24123743/s1. Figure S1: ^1^H NMR spectrum of SC5A (DMSO-*d*_6_, 400 MHz, 25 °C). Figure S2: ^1^H NMR spectrum of SAC4A (DMSO-*d*_6_, 400 MHz, 25 °C). Figure S3: ^1^H NMR spectrum of SAC5A (DMSO-*d*_6_, 400 MHz, 25 °C). Figure S4: ^1^H NMR spectrum of QAAC4A (DMSO-*d*_6_, 400 MHz, 25 °C). Figure S5: Fluorescence spectra produced by dye after adding calixarene. Figure S6: Fluorescence spectra produced by sensor units after adding pesticides. Figure S7: Results of semi-quantitative experiments with sensor array based on different concentrations of bendazone. Figure S8: Experimental results of mixed pesticide sensor array based on thiamethoxam and imidacloprid. Table S1: The training matrix of fluorescence response patterns of different pesticides. Table S2: The training matrix of fluorescence response patterns of 20 blind samples. Table S3: Comparison with other sensor arrays for pesticide discrimination. Table S4: The training matrix of fluorescence response patterns of different concentrations of imazalil. Table S5: The training matrix of fluorescence response patterns of different concentrations of bentazone. Table S6: The training matrix of fluorescence response patterns of mixtures of rimsulfuron and nicosulfuron. Table S7: The training matrix of fluorescence response patterns of mixtures of thiamethoxam and imidacloprid. Table S8: The training matrix of fluorescence response patterns in the presence of 10% soil extract. Table S9: The training matrix of fluorescence response patterns in the presence of 20% soil extract [38,39,40,41,42,43,44,45].
