# Supplementary material for: Calixarene-Based Supramolecular Sensor Array for Pesticide Discrimination
Source: Sensors (Basel). 2024 Jun 8;24(12):3743. doi: 10.3390/s24123743 (PMC11207328; doi:10.3390/s24123743)
Supplement: Supplementary file 1 [file sensors-24-03743-s001.zip › sensors-3035789-supplementary.pdf]

Supplementary Information

For

Calixarene-Based Supramolecular Sensor Array for Pesticide Discrimination

Yeye Chen †, Jia-Hong Tian †, Han-Wen Tian, Rong Ma, Ze-Han Wang, Yu-Chen Pan, Xin-Yue Hu and Dong-Sheng Guo *

College of Chemistry, State Key Laboratory of Elemento-Organic Chemistry, Key Laboratory of Functional Polymer Materials (Ministry of Education), Frontiers Science Center for New Organic Matter, Collaborative Innovation Center of Chemical Science and Engineering, Nankai University, Tianjin 300071, China

* Correspondence: dshguo@nankai.edu.cn

† These authors have contributed equally to this work.

Table of Contents

1. Syntheses of calixarenes 3

2. Supporting information and raw data 8

1. Syntheses of calixarenes

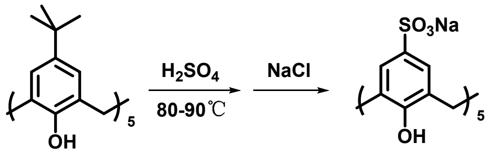

SC5A

Scheme S1. The synthetic route of sulfonatocalix[5]arene (SC5A).

SC5A was synthesized as reported [1]. The direct reaction of *p-tert*-butylcalix[5]arene (1.0 g, 1.23 mmol) with H_2SO_4 (98%, 10 mL) at 80–90 °C for 4 h and periodic checks of the reaction mixture solubility in water, resulting in a dark solution in which most of the calixarene had dissolved. The mixture was quenched in ice (10 g) and then filtered off. The addition of 0.25 g NaCl to the filtrate allowed for a slow reaction, depositing the product as a colorless prism, which was dried by vacuum.

^{1}H NMR (400 MHz, DMSO-*d*6) δ 7.50 (s, 10H, Ar-H), 3.82 (s, 10H, Ar-CH2-Ar) ppm.

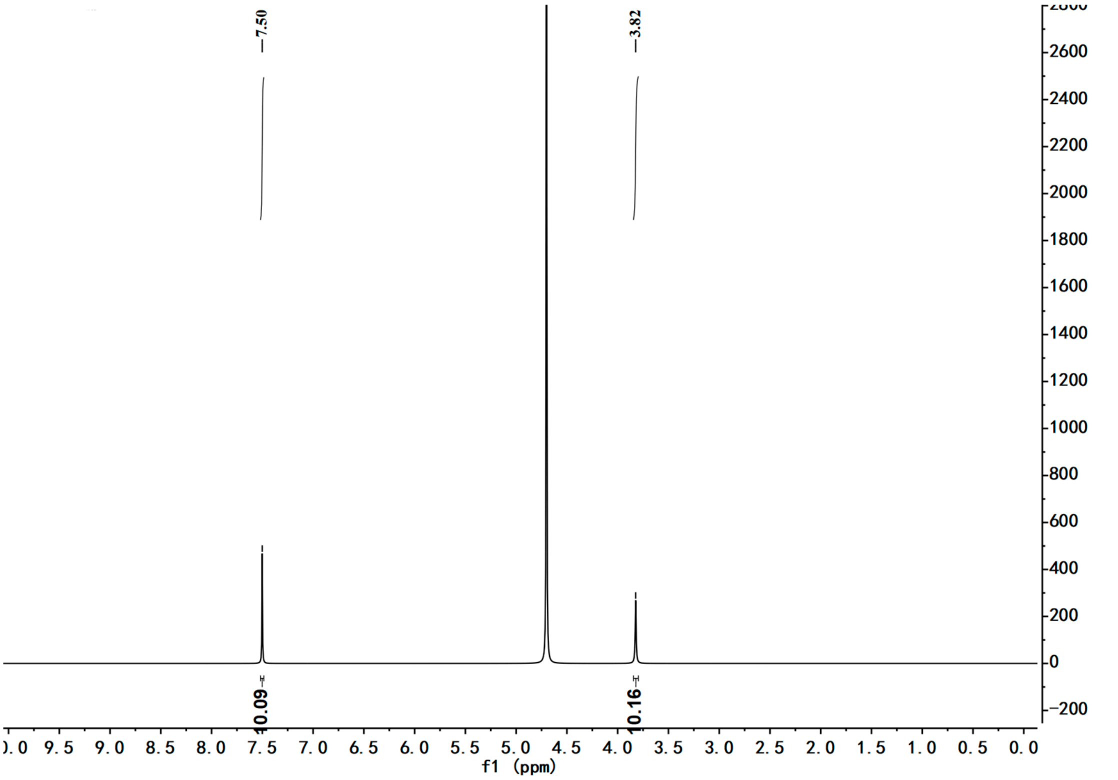

Figure S1. ^{1}H NMR spectrum of SC5A (DMSO-*d*6, 400 MHz, 25 °C).

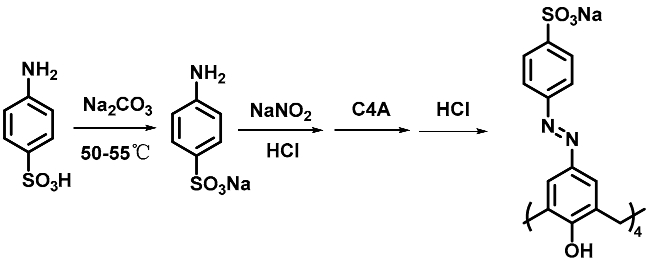

SAC4A

Scheme S2. The synthetic route of sulfonated azocalix[4]arene (SAC4A).

SAC4A was synthesized as reported [2]. Sulfanilic acid (1.732 g, 10 mmol) was dissolved in water (10 mL) containing sodium carbonate (0.518 g, 5 mmol) at 50–55 °C. A solution of $NaNO_2$ (0.702 g, 10 mmol) in water (10 mL) was added to the solution, and then this mixture was added slowly to the concentrated HCl (4 mL) at 0–5 °C for 30 min and further stirred for 1.5 h at this temperature to form 4-sulfobenzenediazonium chloride salt. The obtained solution was added slowly into a solution of 25,26,27,28-tetrahydroxycalix[4]arene (C4A, 1.000 g, 2.36 mmol) and sodium acetate trihydrate (4.080 g, 30 mmol) in MeOH-DMF (26 mL, 5:8, v:v) to obtain a red suspension. The mixture was stirred in an ice bath for 2 h more, and then transferred to room temperature for another 2 h. The solvent was evaporated, and the residue was recrystallized with water/methanol (1:1, v:v). Then, by filtration, the pure product was obtained.

^{1}H NMR (400 MHz, DMSO-*d*6) *δ* 7.77 (s, 8H, calix-Ar-H), 7.73-7.67 (m, 16H, Ar-H), 4.38 and 3.67 (s, 8H, Ar-CH_2-Ar) ppm.

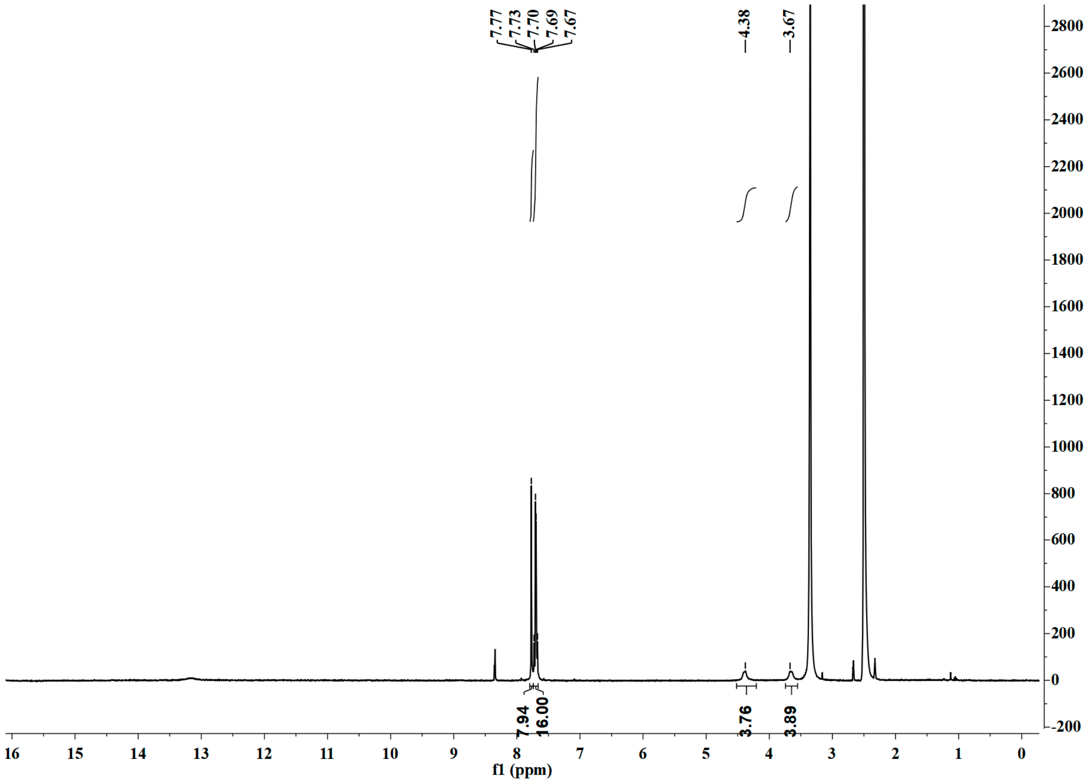

Figure S2. ^{1}H NMR spectrum of SAC4A (DMSO-*d*6, 400 MHz, 25 °C).

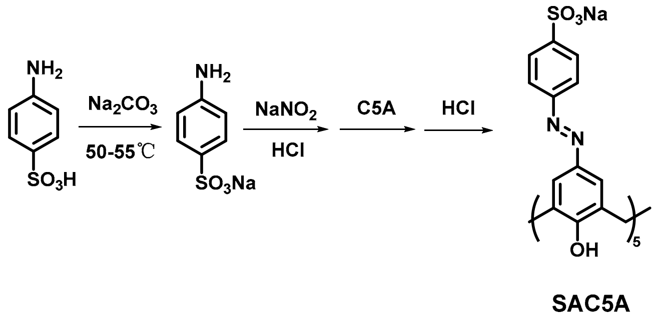

Scheme S3. The synthetic route of sulfonated azocalix[5]arene (SAC5A).

SAC5A was synthesized as reported [3]. Sulfanilic acid (1.732 g, 10 mmol) was

dissolved in water (10 mL) containing sodium carbonate (0.518 g, 5 mmol) at 50–55 °C. A solution of $NaNO_2$ (0.702 g, 10 mmol) in water (10 mL) was added to the above solution, and then this mixture was added slowly to the concentrated HCl (4 mL) at 0–5 °C for 30 min and further stirred at this temperature for 1.5 h to obtain 4-sulfobenzenediazonium chloride salt. The obtained solution was slowly added into a solution of 25,26,27,28-tetrahydroxycalix[5]arene (C5A, 1.1 g, 2.0 mmol) and sodium acetate trihydrate (4.08 g, 30 mmol) in MeOH-DMF (26 mL, 5:8, v:v) to obtain a red suspension. The red reactant was allowed to couple for 2 h more in an ice bath, then acidified by 150 mL of aqueous HCl (0.25%) and warmed at 60 °C for 30 min to produce the reagent as a reddish viscous solid. The residue was recrystallized with water/methanol (60 mL, 1:1, v:v), then the solution was cooled, filtered, and dried to obtain a reddish solid of SAC5A in a quantitative yield.

^{1}H NMR (400 MHz, DMSO-d_6) δ 7.81 (s, 10H, calix-Ar-H), 7.77-7.71 (m, 20H, Ar-H), 3.96 (s, 10H, Ar-CH_2-Ar) ppm.

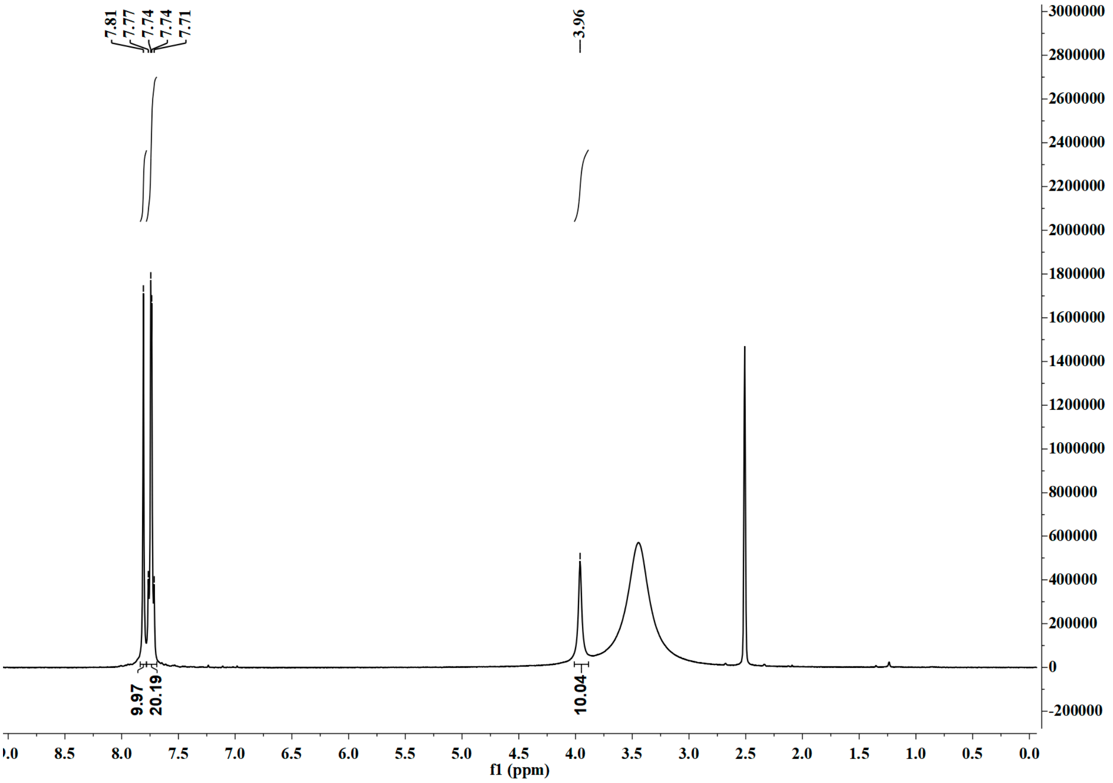

Figure S3. ^{1}H NMR spectrum of SAC5A (DMSO-d_6, 400 MHz, 25 °C).

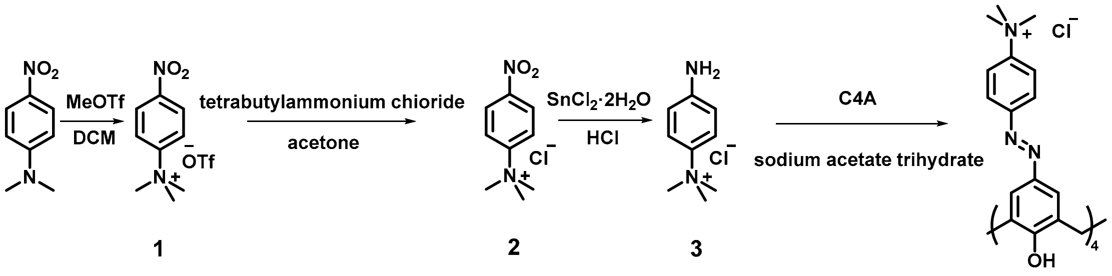

Scheme S4. The synthetic route of quaternary-ammonium-modified azocalix[4]arene (QAAC4A).

QAAC4A was synthesized as reported [4]. General procedure for the synthesis of QAAC4A: *N*, *N*-dimethyl-*p*-nitroaniline (2.1 mmol) was dissolved in 8.0 mL of dichloromethane (DCM), and the solution was cooled to 0 °C. Methyl trifluoromethanesulfonate (MeOTf, 1.0 mL, 4.2 mmol) was slowly added. After 2 h of reaction, a pale-yellow solid was obtained by filtration, named compound **1**. Compound **1** (2.0 mmol) was dissolved in 15.0 mL of acetone, and tetrabutylammonium chloride (2.1 mmol) was added to form a white precipitate, which was washed three times by filtration with acetone to give compound **2**. Compound **2** (3.0 mmol) was dissolved in 5.0 mL of concentrated hydrochloric acid and stannous chloride dihydrate (21.0 mmol). The solution was stirred overnight and then adjusted with NaOH to pH 7. After filtration, the filtrate was spin-dried to give a white solid, which was added to 5.0 mL of methanol. After stirring for 15 min, compound **3** was obtained by filtration. Compound **3** (6.6 mmol) and concentrated hydrochloric acid (1.1 mL) were added to 15.0 mL of water at 0–5 °C. A solution of sodium nitrite (7.2 mmol) in 5 mL of water was slowly added to compound **3** and then stirred for 30 min at 0–5 °C. The resulting solution was slowly added to a solution of MeOH-DMF (26 mL, 5:8, v:v) of 25,26,27,28-tetrahydroxycup[4]aromatic (C4A, 1.6 mmol) and sodium acetate trihydrate (19.8 mmol). After stirring for 2.5 h at room temperature, the solvent was removed by rotary evaporation. The residue was recrystallized from water/acetone, and the solution was then filtered and dried to obtain QAAC4A as a red solid in quantitative yield.

^{1}H NMR (400 MHz, DMSO-d_6) δ 8.09 (d, 8H, J = 8.0 Hz, Ar-H), 7.93 (d, 8H, J = 8.0 Hz, Ar-H), 7.80 (s, 8H, calix-H), 4.43-3.68 (m, 8H, Ar-CH$_2$-H), 3.63 (s, 36H, CH$_3$) ppm.

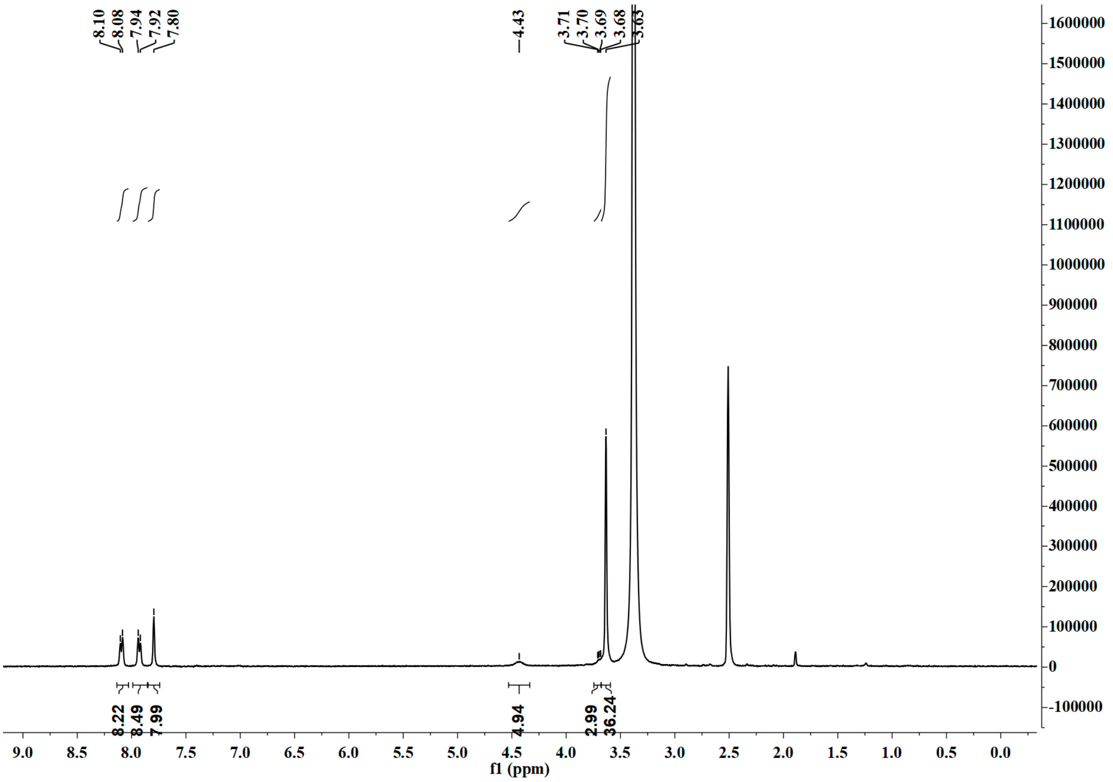

Figure S4. ^{1}H NMR spectrum of QAAC4A (DMSO-d_6, 400 MHz, 25 °C).

2. Supporting information and raw data

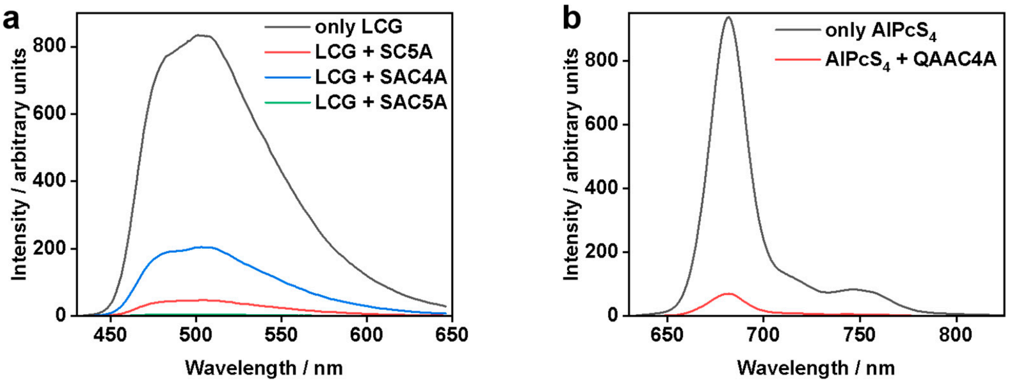

Figure S5. Fluorescence spectra produced by dye after adding calixarene. (a) Fluorescence emission spectra of LCG in the presence of SC5A, SAC4A, and SAC5A, λ_{ex} = 365 nm. (b) Fluorescence emission spectra of $AlPcS_4$ in the presence of QAAC4A, λ_{ex} = 610 nm. Due to the utilization of multiple instruments of the same model in this study, the initial absolute fluorescence intensity may vary across different figures.

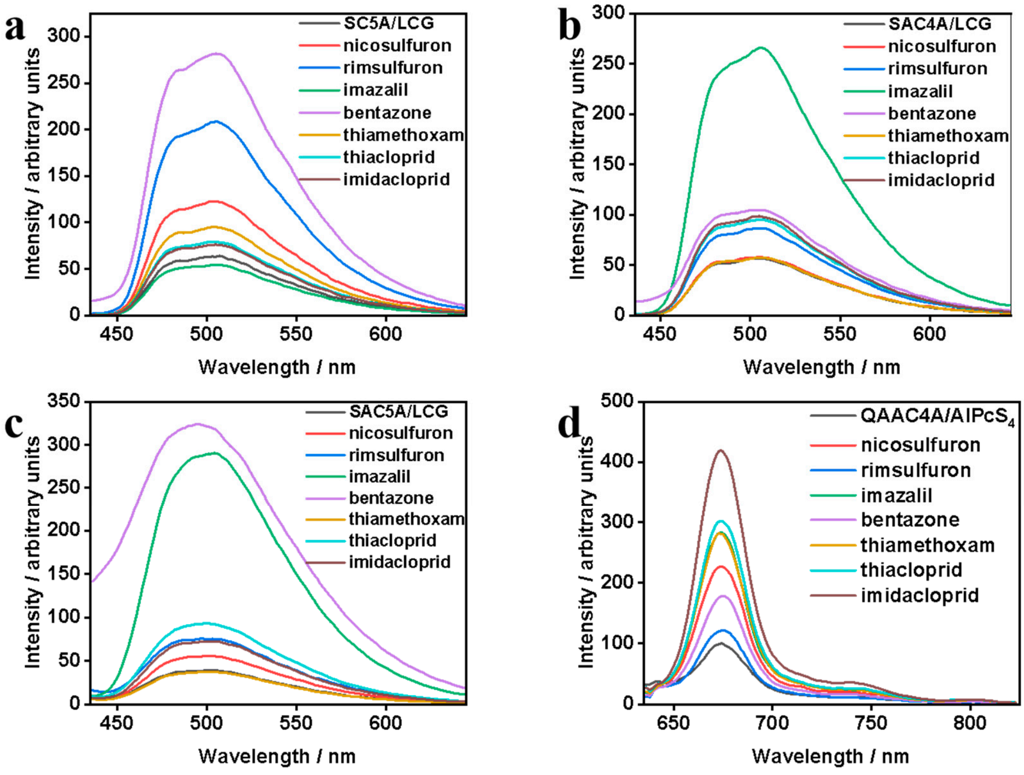

Figure S6. Fluorescence spectra produced by sensor units after adding pesticides. Competitive fluorescence titration of (a) SC5A/LCG (1.0/1.0 μM), (b) SAC4A/LCG (1.0/1.0 μM), (c) SAC5A/LCG (1.0/1.0 μM), and (d) QAAC4A/$AlPcS_4$ (1.0/1.0 μM) with different pesticides (13.0 μg mL^{-1} for each pesticide) at 25 °C. λ_{ex} = 365 nm (a-c), λ_{ex} = 610 nm (d). Due to the utilization of multiple instruments of the same model in this study, the initial absolute fluorescence intensity may vary across different figures.

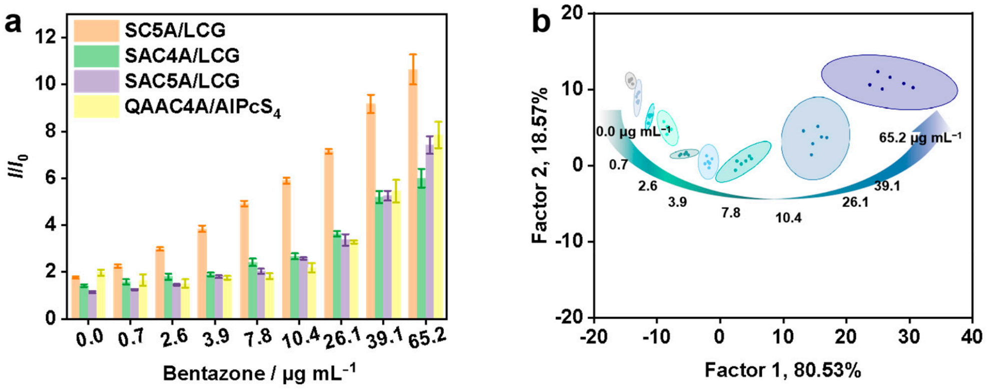

Figure S7. Results of semi-quantitative experiments with sensor array based on different concentrations of bendazone. (a) Fluorescence response patterns and (b) canonical score plot for the detection of bentazone from 0–65.2 μg mL^{-1} (n = 6). Addition of solvent without bendazone resulted in an increase in optical path length, leading to a slight fluorescence response.

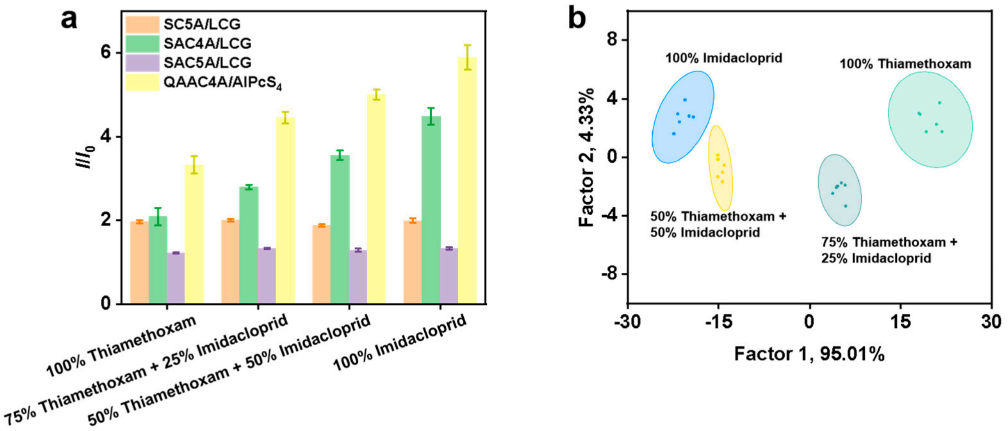

Figure S8. Experimental results of mixed pesticide sensor array based on thiamethoxam and imidacloprid. (a) Fluorescence response patterns and (b) canonical score plot for mixtures (0.5 mg mL^{-1} for total concentration) of thiamethoxam and imidacloprid (n = 6).

Table S1. The training matrix of fluorescence response patterns of different pesticides.

Pesticide	SC5A/LCG	SAC4A/LCG	SAC5A/LCG	QAAC4A/AlPcS$_4$
Nicosulfuron	2.29	2.95	1.29	4.55
Nicosulfuron	2.21	3.22	1.29	4.55
Nicosulfuron	2.19	3.01	1.24	4.33
Nicosulfuron	2.13	3.13	1.25	4.30
Nicosulfuron	2.24	3.14	1.26	4.27
Nicosulfuron	2.22	2.53	1.32	4.23
Rimsulfuron	3.07	1.74	1.32	3.76
Rimsulfuron	3.08	1.81	1.30	3.59

Rimsulfuron	3.02	1.89	1.30	3.60
Rimsulfuron	3.05	1.83	1.29	3.66
Rimsulfuron	2.97	1.99	1.29	3.79
Rimsulfuron	3.19	1.74	1.35	3.56
Imazalil	3.23	12.52	4.75	5.42
Imazalil	3.20	12.30	4.38	5.51
Imazalil	3.42	12.79	4.51	5.39
Imazalil	3.13	12.19	3.95	5.08
Imazalil	3.08	11.79	3.61	5.72
Imazalil	2.90	10.46	3.55	5.39
Bentazone	7.78	3.32	3.40	4.29
Bentazone	7.82	3.12	3.21	4.02
Bentazone	8.05	3.38	3.14	4.25
Bentazone	7.40	3.30	2.77	3.97
Bentazone	6.99	3.13	2.88	4.10
Bentazone	7.72	3.03	2.92	4.10
Thiamethoxam	1.83	1.32	1.18	4.49
Thiamethoxam	1.81	1.30	1.19	4.60
Thiamethoxam	1.74	1.35	1.20	4.65
Thiamethoxam	1.74	1.32	1.33	4.54
Thiamethoxam	1.76	1.30	1.22	4.68
Thiamethoxam	1.78	1.29	1.21	4.77
Thiacloprid	2.06	2.79	1.29	5.64
Thiacloprid	2.00	2.55	1.23	5.85
Thiacloprid	1.92	2.55	1.19	5.96
Thiacloprid	2.02	2.51	1.22	5.98
Thiacloprid	1.99	2.71	1.28	5.84
Thiacloprid	1.98	2.58	1.25	5.70
Imidacloprid	1.72	2.12	1.29	5.70
Imidacloprid	1.77	2.06	1.23	5.57
Imidacloprid	1.74	2.11	1.19	5.68
Imidacloprid	1.78	2.15	1.25	5.89
Imidacloprid	1.75	2.19	1.22	5.78
Imidacloprid	1.72	2.20	1.27	5.49

Table S2. The training matrix of fluorescence response patterns of 20 blind samples.

Sample No.	SC5A/LCG	SAC4A/LCG	SAC5A/LCG	QAAC4A/ $AlPcS_4$	Discrimination	Verification
1	3.03	12.03	4.24	5.57	Imazalil	Yes
2	2.18	2.99	1.27	4.28	Nicosulfuron	Yes
3	1.78	1.30	1.26	4.55	Thiamethoxam	Yes
4	7.66	3.17	2.84	4.09	Bentazone	Yes
5	1.79	1.32	1.23	4.59	Thiacloprid	Yes

6	7.54	3.11	2.97	4.01	Bentazone	Yes
7	2.03	2.59	1.26	5.93	Thiacloprid	Yes
8	7.37	3.15	3.13	4.09	Bentazone	Yes
9	3.09	1.79	1.31	3.57	Rimsulfuron	Yes
10	7.43	3.32	3.07	4.20	Bentazone	Yes
11	2.23	3.11	1.28	4.49	Nicosulfuron	Yes
12	1.74	2.16	1.26	5.56	Imidacloprid	Yes
13	3.22	12.66	4.39	5.42	Imazalil	Yes
14	2.24	2.78	1.27	4.29	Nicosulfuron	Yes
15	7.89	3.29	3.01	4.15	Bentazone	Yes
16	2.99	11.78	4.43	5.33	Imazalil	Yes
17	3.15	11.95	3.77	5.29	Imazalil	Yes
18	1.79	2.19	1.26	5.81	Blank Area	No
19	3.11	1.83	1.30	3.63	Rimsulfuron	Yes
20	3.12	1.89	1.31	3.56	Rimsulfuron	Yes

Table S3. Comparison with other sensor arrays for pesticide discrimination.

Method	Material	Number of tested pesticides	Detection limits	Accuracy for blind samples	Ref.
Chemiluminescent sensor array	Silver nanoparticles	5	24 μg mL^{-1}	95%	[5]
Colorimetric sensor array	Gold nanoparticles	5	120 ng mL^{-1}	-	[6]
Fluorescent sensor array	Macrocyclic receptors	5	10 μM	-	[7]
Colorimetric sensor array	Nanozymes	8	10 μM	-	[8]
Fluorescent sensor array	Macrocyclic receptors	7	13 μg mL^{-1}	95%	This work

Table S4. The training matrix of fluorescence response patterns of different concentrations of imazalil.

Concentration/μg mL^{-1}	SC5A/LCG	SAC4A/LCG	SAC5A/LCG	QAAC4A/AlPcS$_4$
0	1.84	1.67	1.25	2.27
0	1.85	1.69	1.15	2.18
0	1.86	1.66	1.17	2.07
0	1.85	1.59	1.28	2.19
0	1.79	1.57	1.23	2.01
0	1.83	1.58	1.20	2.22
0.13	2.21	2.06	1.33	2.05
0.13	2.22	2.05	1.31	1.94

0.13	2.08	2.24	1.24	2.06
0.13	2.20	2.05	1.29	2.05
0.13	2.18	2.06	1.27	2.10
0.13	2.12	2.30	1.30	2.25
0.65	2.34	3.03	1.44	2.21
0.65	2.22	3.11	1.39	2.14
0.65	2.27	2.95	1.30	2.15
0.65	2.42	3.19	1.52	2.14
0.65	2.34	3.12	1.41	2.27
0.65	2.31	3.04	1.39	2.14
0.98	2.47	3.76	1.50	2.54
0.98	2.62	3.94	1.52	2.49
0.98	2.66	3.70	1.46	2.58
0.98	2.58	3.77	1.42	2.30
0.98	2.61	3.93	1.50	2.28
0.98	2.62	3.74	1.45	2.51
1.30	2.77	4.47	1.64	2.72
1.30	2.93	4.35	1.59	2.85
1.30	2.86	4.36	1.64	2.65
1.30	2.75	4.37	1.57	2.80
1.30	2.82	4.44	1.73	2.75
1.30	2.93	4.47	1.65	2.78
2.61	3.04	5.95	1.87	3.06
2.61	3.11	6.19	1.89	2.91
2.61	3.07	6.02	1.89	2.93
2.61	3.10	5.94	1.98	2.93
2.61	2.96	5.50	1.85	2.69
2.61	3.20	5.50	1.94	3.19
5.22	3.62	7.57	2.41	3.25
5.22	3.30	7.48	2.33	3.15
5.22	3.17	7.42	2.11	2.91
5.22	3.40	7.68	2.20	3.11
5.22	3.39	7.21	2.32	3.03
5.22	3.41	7.48	2.13	3.24
7.82	3.54	9.32	2.55	3.98
7.82	3.53	9.57	2.47	4.09
7.82	3.55	9.69	2.42	3.95
7.82	3.52	9.55	2.28	4.09
7.82	3.81	9.68	2.48	3.55
7.82	3.51	9.47	2.30	3.81
13.04	4.01	11.22	3.28	4.38
13.04	4.01	11.68	3.34	4.65
13.04	4.23	12.23	3.57	4.12
13.04	4.13	11.77	3.29	3.96

13.04	4.17	12.04	3.75	3.91
13.04	4.54	11.81	3.67	4.23
19.57	4.26	13.61	4.75	5.42
19.57	4.25	12.66	4.94	5.10
19.57	4.14	13.53	4.26	5.20
19.57	4.34	13.66	4.44	5.00
19.57	4.40	14.14	4.87	5.42
19.57	4.53	14.08	4.39	5.71

Table S5. The training matrix of fluorescence response patterns of different concentrations of bentazone.

Concentration/μg mL^{-1}	SC5A/LCG	SAC4A/LCG	SAC5A/LCG	QAAC4A/AlPcS$_4$
0	1.77	1.37	1.12	2.02
0	1.74	1.42	1.10	1.94
0	1.68	1.36	1.12	1.90
0	1.80	1.42	1.15	2.13
0	1.79	1.41	1.14	2.07
0	1.80	1.52	1.22	1.76
0.65	2.17	1.78	1.26	1.81
0.65	2.31	1.46	1.25	2.03
0.65	2.19	1.54	1.23	1.66
0.65	2.25	1.52	1.25	1.41
0.65	2.34	1.60	1.23	1.57
0.65	2.25	1.58	1.30	1.43
2.61	2.90	1.90	1.49	1.36
2.61	2.89	1.71	1.48	1.38
2.61	3.01	1.92	1.41	1.32
2.61	3.00	1.67	1.45	1.55
2.61	3.01	1.95	1.44	1.76
2.61	3.08	1.66	1.47	1.69
3.91	3.59	1.82	1.75	1.78
3.91	3.91	1.89	1.82	1.75
3.91	3.94	1.93	1.75	1.64
3.91	3.83	2.03	1.80	1.86
3.91	3.99	1.88	1.91	1.73
3.91	3.82	1.83	1.80	1.77
7.83	4.93	2.42	2.14	1.71
7.83	4.74	2.56	1.98	1.67
7.83	5.03	2.63	2.15	1.85
7.83	4.83	2.34	1.88	1.81
7.83	4.98	2.33	2.02	1.91
7.83	5.03	2.22	2.05	2.00
10.43	5.77	2.48	2.56	2.29
10.43	6.05	2.60	2.64	2.48

10.43	5.78	2.70	2.50	2.02
10.43	5.97	2.85	2.58	2.29
10.43	5.99	2.76	2.58	2.01
10.43	5.82	2.63	2.61	2.00
26.09	7.18	3.44	3.28	3.30
26.09	6.97	3.59	3.17	3.33
26.09	7.14	3.61	3.05	3.30
26.09	7.22	3.66	3.43	3.37
26.09	7.15	3.70	3.62	3.25
26.09	7.24	3.78	3.67	3.18
39.13	9.24	5.42	5.46	5.39
39.13	9.24	5.02	5.12	4.78
39.13	8.57	4.84	5.04	5.11
39.13	9.37	5.08	5.03	5.78
39.13	9.70	5.41	5.43	6.13
39.13	8.92	5.46	5.44	5.52
65.22	10.46	6.35	7.52	7.81
65.22	11.39	6.47	7.59	8.87
65.22	11.48	6.23	8.02	8.01
65.22	10.01	5.75	6.93	7.38
65.22	10.46	5.73	7.25	7.34
65.22	10.08	5.50	7.20	7.69

Table S6. The training matrix of fluorescence response patterns of mixtures of rimsulfuron and nicosulfuron.

Mixtures	SC5A/LCG	SAC4A/LCG	SAC5A/LCG	QAAC4A/AlPcS$_4$
100% Rimsulfuron	10.97	8.73	3.83	1.56
100% Rimsulfuron	10.83	9.01	3.66	1.63
100% Rimsulfuron	10.68	8.33	3.35	1.63
100% Rimsulfuron	11.23	9.19	3.65	1.62
100% Rimsulfuron	11.20	8.83	3.19	1.65
100% Rimsulfuron	11.50	8.28	3.48	1.58
75% Rimsulfuron + 25% Nicosulfuron	10.34	6.36	2.79	1.50
75% Rimsulfuron + 25% Nicosulfuron	9.90	6.12	2.62	1.53
75% Rimsulfuron + 25% Nicosulfuron	9.90	6.11	2.64	1.51
75% Rimsulfuron + 25% Nicosulfuron	9.35	6.15	2.59	1.45
75% Rimsulfuron + 25% Nicosulfuron	9.83	6.70	2.63	1.49
75% Rimsulfuron + 25% Nicosulfuron	9.40	6.38	2.58	1.51

50% Rimsulfuron + 50% Nicosulfuron	7.92	5.04	1.86	1.54
50% Rimsulfuron + 50% Nicosulfuron	7.04	4.56	1.74	1.54
50% Rimsulfuron + 50% Nicosulfuron	7.44	4.72	1.81	1.59
50% Rimsulfuron + 50% Nicosulfuron	7.53	4.62	1.74	1.48
50% Rimsulfuron + 50% Nicosulfuron	7.80	4.45	1.88	1.73
50% Rimsulfuron + 50% Nicosulfuron	7.25	4.35	1.87	1.62
25% Rimsulfuron + 75% Nicosulfuron	5.23	3.02	1.50	2.54
25% Rimsulfuron + 75% Nicosulfuron	5.06	3.13	1.57	2.48
25% Rimsulfuron + 75% Nicosulfuron	4.88	3.31	1.56	2.63
25% Rimsulfuron + 75% Nicosulfuron	4.76	3.01	1.49	2.51
25% Rimsulfuron + 75% Nicosulfuron	4.67	3.07	1.50	2.58
25% Rimsulfuron + 75% Nicosulfuron	4.92	2.89	1.51	2.48
100% Nicosulfuron	2.86	2.25	1.41	3.85
100% Nicosulfuron	2.86	2.31	1.38	3.75
100% Nicosulfuron	2.77	2.16	1.34	3.78
100% Nicosulfuron	2.75	2.29	1.39	3.51
100% Nicosulfuron	2.72	2.21	1.34	3.56
100% Nicosulfuron	2.81	2.01	1.36	3.71

Table S7. The training matrix of fluorescence response patterns of mixtures of thiamethoxam and imidacloprid.

percentage	SC5A/LCG	SAC4A/LCG	SAC5A/LCG	QAAC4A/AlPcS$_4$
100% Thiamethoxam	1.93	1.80	1.22	3.37
100% Thiamethoxam	1.96	1.89	1.20	3.47
100% Thiamethoxam	2.04	2.29	1.24	3.04
100% Thiamethoxam	1.93	2.27	1.20	3.42
100% Thiamethoxam	1.98	2.24	1.24	3.11
100% Thiamethoxam	1.97	2.05	1.23	3.56
75% Thiamethoxam + 25% Imidacloprid	1.97	2.84	1.32	4.54
75% Thiamethoxam + 25% Imidacloprid	2.00	2.82	1.37	4.40

75% Thiamethoxam + 25% Imidacloprid	2.00	2.78	1.33	4.62
75% Thiamethoxam + 25% Imidacloprid	2.03	2.75	1.32	4.33
75% Thiamethoxam + 25% Imidacloprid	2.05	2.73	1.31	4.55
75% Thiamethoxam + 25% Imidacloprid	1.96	2.86	1.32	4.27
50% Thiamethoxam + 50% Imidacloprid	1.85	3.37	1.25	5.11
50% Thiamethoxam + 50% Imidacloprid	1.86	3.53	1.28	5.08
50% Thiamethoxam + 50% Imidacloprid	1.84	3.58	1.26	4.96
50% Thiamethoxam + 50% Imidacloprid	1.90	3.53	1.31	5.14
50% Thiamethoxam + 50% Imidacloprid	1.90	3.70	1.35	4.88
50% Thiamethoxam + 50% Imidacloprid	1.91	3.63	1.26	4.88
100% Imidacloprid	1.94	4.25	1.31	6.19
100% Imidacloprid	2.03	4.63	1.30	5.73
100% Imidacloprid	1.95	4.57	1.35	6.14
100% Imidacloprid	1.95	4.45	1.35	5.57
100% Imidacloprid	2.02	4.27	1.32	5.58
100% Imidacloprid	2.06	4.75	1.35	6.14

Table S8. The training matrix of fluorescence response patterns in the presence of 10% soil extract.

Pesticide	SC5A/LCG	SAC4A/LCG	SAC5A/LCG	QAAC4A/AlPcS$_4$
Nicosulfuron	4.07	3.18	1.45	5.34
Nicosulfuron	3.98	2.94	1.41	5.56
Nicosulfuron	3.88	3.08	1.48	5.58
Nicosulfuron	3.94	2.79	1.38	5.38
Nicosulfuron	4.03	3.06	1.42	5.53
Nicosulfuron	3.79	3.15	1.35	5.55
Rimsulfuron	4.38	3.07	1.36	5.69
Rimsulfuron	4.41	3.21	1.39	5.69
Rimsulfuron	4.37	3.11	1.33	5.33
Rimsulfuron	4.42	3.07	1.40	5.49
Rimsulfuron	4.45	3.05	1.39	4.75
Rimsulfuron	4.27	3.05	1.35	5.18
Imazalil	4.74	12.15	4.19	8.26
Imazalil	4.93	12.46	4.75	8.43

Imazalil	4.86	12.24	4.39	8.78
Imazalil	4.95	11.33	4.47	7.90
Imazalil	4.99	11.48	4.33	8.19
Imazalil	4.80	11.67	4.64	7.63
Bentazone	6.39	3.92	2.89	4.76
Bentazone	6.36	3.91	2.82	4.71
Bentazone	6.51	4.10	3.02	4.45
Bentazone	6.43	3.77	2.79	4.79
Bentazone	6.41	4.10	3.05	5.00
Bentazone	6.27	3.84	2.81	4.39
Thiamethoxam	2.81	2.61	1.34	7.73
Thiamethoxam	2.97	2.71	1.41	8.10
Thiamethoxam	2.82	2.60	1.44	7.80
Thiamethoxam	2.79	2.74	1.40	7.44
Thiamethoxam	2.88	2.63	1.33	7.92
Thiamethoxam	2.91	2.65	1.37	7.20
Thiacloprid	2.95	4.25	1.35	8.33
Thiacloprid	2.79	4.46	1.42	8.38
Thiacloprid	2.96	4.57	1.42	7.52
Thiacloprid	2.95	4.36	1.42	7.73
Thiacloprid	2.94	4.29	1.39	8.07
Thiacloprid	2.88	4.43	1.39	7.49
Imidacloprid	2.86	3.80	1.37	7.33
Imidacloprid	2.98	3.76	1.37	7.34
Imidacloprid	3.03	3.62	1.43	8.05
Imidacloprid	3.06	3.76	1.38	7.17
Imidacloprid	2.93	3.81	1.39	7.95
Imidacloprid	3.00	3.81	1.36	7.21

Table S9. The training matrix of fluorescence response patterns in the presence of 20% soil extract.

Pesticide	SC5A/LCG	SAC4A/LCG	SAC5A/LCG	QAAC4A/AlPcS$_4$
Nicosulfuron	4.39	3.38	1.63	16.35
Nicosulfuron	4.30	3.26	1.65	15.61
Nicosulfuron	4.35	3.00	1.64	15.15
Nicosulfuron	4.45	3.11	1.66	16.20
Nicosulfuron	4.45	2.98	1.60	15.23
Nicosulfuron	4.32	3.28	1.63	16.22
Rimsulfuron	4.80	3.51	1.72	15.42
Rimsulfuron	4.85	3.71	1.73	16.23
Rimsulfuron	4.84	3.53	1.74	15.73
Rimsulfuron	4.78	3.43	1.79	16.24
Rimsulfuron	4.80	3.92	1.75	15.63
Rimsulfuron	4.64	3.87	1.70	16.60

Imazalil	5.10	11.14	3.98	17.09
Imazalil	5.21	11.12	4.27	16.98
Imazalil	5.26	10.99	4.08	16.55
Imazalil	4.98	11.39	4.14	17.28
Imazalil	5.37	11.26	3.94	16.36
Imazalil	5.15	10.70	4.28	17.32
Bentazone	5.74	4.19	3.42	11.92
Bentazone	5.49	4.21	3.71	12.81
Bentazone	5.63	4.12	3.51	12.76
Bentazone	5.71	4.40	3.59	13.33
Bentazone	5.56	4.44	3.49	12.67
Bentazone	5.77	4.35	3.52	11.79
Thiamethoxam	3.34	3.47	1.58	15.17
Thiamethoxam	3.39	3.56	1.53	16.16
Thiamethoxam	3.39	3.22	1.54	15.02
Thiamethoxam	3.48	3.59	1.56	15.03
Thiamethoxam	3.46	3.33	1.54	16.31
Thiamethoxam	3.49	3.33	1.55	15.17
Thiacloprid	3.66	5.65	1.75	16.52
Thiacloprid	3.68	6.01	1.73	16.80
Thiacloprid	3.70	5.89	1.78	18.29
Thiacloprid	3.76	6.03	1.74	16.42
Thiacloprid	3.77	5.65	1.69	15.03
Thiacloprid	3.77	5.86	1.74	14.90
Imidacloprid	3.33	4.55	1.59	13.01
Imidacloprid	3.60	4.37	1.56	14.38
Imidacloprid	3.48	4.45	1.57	14.18
Imidacloprid	3.63	4.82	1.59	14.89
Imidacloprid	3.59	4.68	1.54	14.86
Imidacloprid	3.58	4.63	1.50	12.22

References

[1] Gutsche, C. D.; Bauer, L. J. Calixarenes. 13. The conformational properties of calix[4]arenes, calix[6]arenes, calix[8]arenes, and oxacalixarenes. *J. Am. Chem. Soc.* **1985**, 107, 6052–6059. https://doi.org/10.1021/ja00307a038.

[2] Lu, L.; Zhu, S.; Liu, X.; Xie, Z.; Yan, X. Highly selective chromogenic ionophores for the recognition of chromium(III) based on a water-soluble azocalixarene derivative. *Anal. Chim. Acta* **2005**, 535, 183–187. https://doi.org/10.1016/j.aca.2004.11.059.

[3] Yue, Y.-X.; Zhang, Z.; Wang, Z.-H.; Ma, R.; Chen, M.-M.; Ding, F.; Li, H.-B.; Li, J.-J.; Shi, L.; Liu, Y.; Guo, D.-S. Promoting tumor accumulation of anticancer drugs by hierarchical carrying of exogenous and endogenous vehicles. *Small Struct.* **2022**, 3, 2200067. https://doi.org/10.1002/sstr.202200067.

[4] Shinkai, S.; Araki, K.; Shibata, J.; Tsugawa, D.; Manabe, O. Diazo-coupling reactions with calix[4]arene. pKa determination with chromophoric azocalix[4]arenes. *Chem. Lett.* **1989**, 18, 931–934. https://doi.org/10.1246/cl.1989.931.

[5] He, Y.; Xu, B.; Li, W.; Yu, H. Silver nanoparticle-based chemiluminescent sensor array for pesticide discrimination. *J. Agric. Food Chem.* 2015, 63, 2930–2934. https://doi.org/10.1021/acs.jafc.5b00671.

[6] Fahimi-Kashani, N.; Hormozi-Nezhad, M. R., Gold-nanoparticle-based colorimetric sensor array for discrimination of organophosphate pesticides. *Anal. Chem.* **2016**, 88, 8099–8106. https://doi.org/10.1021/acs.analchem.6b01616.

[7] Wei, K.-N.; Yang, R.-P.; Huang, S.-Z.; Tao, Z.; Tang, Q.; Huang, Y., Supramolecular fluorescence sensor array based on cucurbit[8]uril complexes used for the detection of multiplex quaternary ammonium pesticides. *J. Agric. Food Chem.* **2023**, 71, 9549–9557. https://doi.org/10.1021/acs.jafc.3c00347.

[8] Kumar, M.; Kaur, N.; Singh, N. Colorimetric nanozyme sensor array based on metal nanoparticle-decorated CNTs for quantification of pesticides in real water and soil samples. *ACS Sustainable Chem. Eng.* **2024**, 12, 728–736. https://doi.org/10.1021/acssuschemeng.3c04153.
